# In-depth analysis of magnetic flux concentration indicator in novel curved slots of multi-tooth dual-PM doubly salient machine

**DOI:** 10.1038/s41598-025-99299-9

**Published:** 2025-04-22

**Authors:** Apirat Siritaratiwat, Pattasad Seangwong, Authumporn Butkaew, Pirat Khunkitti, Warat Sriwannarat

**Affiliations:** 1 Department of Electrical Engineering, Faculty of Engineering, Khon Kaen University, Khon Kaen, 40002 Thailand; 2 Department of Electrical and Computer Engineering, Faculty of Science and Engineering, Kasetsart University Chalermphakiet Sakon Nakhon Province Campus, Sakon Nakhon, 47000 Thailand

**Keywords:** Magnetic flux concentration indicator, Curved slots, Multi-tooth structure, Dual-PM doubly salient machine, Electrical and electronic engineering, Mechanical engineering

## Abstract

Multi-tooth structure in dual-PM doubly salient machine (MT-DDSPM) has discovered large flux leakage with a non-concentrated magnetic flux due to many square teeth and many edges at teeth and poles. To overcome these shortcomings, this paper proposed a novel design of the curved slots applied in the MT-DDSPM. In addition, the magnetic flux concentration indicator (MFCI) was originally formulated to measure the magnetic flux concentration at the stator teeth, focused mainly on the edge and slots. These aimed to optimize the design of curved slots applied in the 12/3/35-pole MT-DDSPM for superior torque production. The electromagnetic performance of the designed MT-DDSPM was deeply investigated using the finite element method in comparison to traditional square-slot design. The invented MT-DDSPM with curved slots revealed the highest MFCI of 25.47 compared to 19.16 obtained with the square slots. The results indicated an enhanced magnetic flux concentration of MT-DDSPM, leading to higher flux linkage and other electromagnetic performances. Especially, the rated torque obtained from the curved-slot design was 66.01% higher than the square one at the same rated current, providing a better profile even at overload current. The minimum ripple torque of 1.35% was found with the curved-slot MT-DDSPM. In conclusion, this work highlighted the superior performance of a novel curved-slot MT-DDSPM and provided a reliable alternative to evaluate the quality of magnetic flux concentration in terms of MFCI, which can be essential for the in-depth analysis of the multi-tooth family of electrical machines and relevant applications.

## Introduction

STATOR permanent magnet (PM) machines have gained widespread attention for several years because of their top-range-quality performance in terms of torque production^1^. Based on its structure, the PM mounted at the stator has outstanding merits of the rotor, such as low weight, small inertia, robustness, and easy thermal management^2^. Moreover, the traditional advantage of the PM machine, such as the absence of the DC-excited coil, has still appeared in the structure of this machine category^3^. The stator PM machines have been favored to operate in several applications, such as renewable generators, electric vehicle motors, maglev trains, and industrial pumps^4–6^. According to the position of PM installation, the categorization of such machines has been divided into three main types i.e., flux-switched PM machine (FSPM) with the PM installed at stator teeth for using wide-speed application, flux-reversal PM machine (FRPM) with the PM mounted at the tip of stator teeth for operating at high-speed velocity and doubly salient PM machines (DSPM) with PM placed at stator yoke for utilizing at low-speed range application^7–10^. The comparative machine capability and their application were introduced in^11^.

The DSPM evolved from the switched reluctance machine provides many advantages, such as a simple structure, good PM protection, high back-electromagnetic force (back-EMF), and high torque density^12^. The operation principle of three-phase DSPM has been continually studied for the last decade to improve the magnetic flux concentration for positive performance production^13^. This machine has been designed to be used in direct-drive and large torque applications because its structure contains a short stack length and wide diameter^14^. Recently, the DSPM has been classified into two new branches for balancing the magnetic flux path to create a symmetric back-EMF waveform, including a hybrid-excited slot-PM machine (HE-SPM) has the PM placed at the opening slot^15–17^. A biased flux PM machine (BFPM) has the PM mounted between stator poles^18,19^. A combination of HE-SPM and BFPM, namely dual-PM double salient machine (DDSPM), was presented to destroy the constraints of torque production and flux regulation quality occurring from the overlap coil winding and the nonadjustable PM flux^20,21^.

The issues of the machinery characteristics from over-magnet usage and narrow space for winding the coil, which suffer harmful torque production of the DSPM family, have been discovered in many research studies^22^. To fix this problem, the split-tooth or multi-tooth technique was proposed to contain the DSPM family. In 2020, the split-tooth HE-SPM indicated high torque characteristics due to the improvement of the fault-tolerant capability^23^. The multi teeth were presented in the outer rotor DDSPM, aiming to improve torque density in 2023. Its stator/rotor pole was considered for low ripple torque. Particularly, this machine showed the analysis of the magnetic flux controlling the working harmonic of the air-flux density for better amplitude than other structures^24^. Modifications were introduced to the DDSPM from conventional to multi-tooth structures. The method of the topology arrangement was described in detail^25^. A comparative study of several DSPM types was currently performed. In^26^, the investigation of the air-gap flux density found that the DDSPM with a multi-tooth structure exhibits higher torque density than the other DSPM structure.

However, these multi-tooth configurations must avoid non-concentrated magnetic flux with unsmooth flow because they have many square teeth and many edges at stator teeth and rotor poles^27^. It results in a messy magnetic flux flow along the square edge, causing a large leakage flux even with the low flux linkage, negatively impacting torque production. This indicates the unpleasant control of the magnetic flux at the stator teeth, focused only on the edge and slot^28,29^. Many research reviews have attempted to address this negative impact, such as evaluating tooth force using skewing configuration, the magnetic flux management utilizing the flux barrier design and tooth geometries optimization by replacing the sharp edges^30,31^. The optimization of the tooth shape is very famous because it provides direct magnetic flux quantification and flexibility in customization compared to other methods. The design of curved slots has been suggested to directly impact the magnetic flux concentration around stator teeth^32^. For instance, the stator and rotor slot in variable-flux PM machines were designed to be curved for a more uniform and smoother magnetic flux effect to better concentrate the flux^33^. Moreover, the geometry of the slotted-rotor teeth of electrical machines was redesigned to improve the magnetic flux concentration^34,35^. However, most research only employed the curved slot design in conventional machine structures.

In this work, the curved slots were innovatively applied in the multi-tooth DDSPM structure. This combines the advantages of the curved slots and the enhanced air-gap flux distribution from the multi-tooth structure. The magnetic flux concentration indicator (MFCI) was originally invented for an in-depth analysis of magnetic flux at the stator teeth. Firstly, primary machine topologies and their stator/rotor pole selection were considered. The parameters of curved slots were optimized to aim the high torque production. The principle of the magnetic equivalent circuit (MEC) was investigated to verify with magnetic flux distribution of the designed curved-slot MT-DDSPM. The other electromagnetic performance, including air-gap flux density, MFCI, flux linkage, back-EMF, total harmonic distortion (THD), torque characteristics and PM demagnetization, curved-slot MT-DDSPM was investigated by the 2-D finite element method (2-D FEM) under no-load, load and overload condition. Finally, the simulation results were compared to the initial and traditional structure for fair constraint.

## Machine topologies selection

### Primary machine topologies

The stator/rotor pole of the MT-DDSPM was initially studied as inspired by combining the basis pole calculation of the doubly salient family and multi-tooth structure of consequent-pole FRPM^8,10^. It can be expressed by

1 $$\left\{ \begin{gathered} N_{s} = 2mk \hfill \\ n = k + 1 \hfill \\ N_{r} = \left( {n \times N_{s} } \right) \pm i \hfill \\ \end{gathered} \right.$$

where* N*_*s*_ is the stator pole number, *N*_*r*_ is the rotor pole number, *m* is the number of phases, *n* is the stator teeth number and k and *i* are the positive integers.

Consideration of the stator/rotor pole combination is essential to achieve suitability for the magnetic flux path, affecting the overall performance of the electrical machine. However, an over-large number of *n* also results in the deterioration of torque production because it produces a useless leakage flux. The possible stator/rotor pole is distributed to express the calculation of the rotor pole numbers, as shown in Table 1.

**Table 1:** Stator/rotor pole of MT-DDSPMs.

*k*	*n*	*m*	Formula	*N*_*s*_	Formula	*N*_*r*_
1	2	3	*2mk*	6	(*n* × *N*_*s*_) − *2*	10
					(*n* × *N*_*s*_) − *1*	11
					(*n* × *N*_*s*_) + *1*	13
					(*n* × *N*_*s*_) + *2*	14
2	3	3	*2mk*	12	(*n* × *N*_*s*_) − *2*	34
					(*n* × *N*_*s*_) − *1*	35
					(*n* × *N*_*s*_) + *1*	37
					(*n* × *N*_*s*_) + *2*	38

While multiple stator/rotor poles are theoretically feasible, the 12/3/35-poles (*k* = 2) structure is selected as a suitable stator/rotor pole because it provides its superior balance of performance as well as flux concentration. This selected design shows better fundamental indicators such as back-EMF, average torque and leakage flux compared to the 6/2/13-poles (*k* = 1) MT-DDSPM, as summarized in Table 2.

**Table 2:** Selection of pole configuration of MT-DDSPMs.

Pole configuration	Back-EMF (V)	Average torque (Nm)	Leakage flux
6/2/13-pole^25^	118.12	19.47	High
12/3/35-pole (selected)	121.04	19.95	Low
12/3/37-pole (alternative)	111.96	19.17	Medium

Nevertheless, the conventional multi-tooth structure suffers from a non-concentrated magnetic flux, affecting the wasted leakage flux due to its square teeth and edges at stator teeth and rotor poles. In this study, curved slots are proposed as a solution to address the issue.

**Fig. 1:**
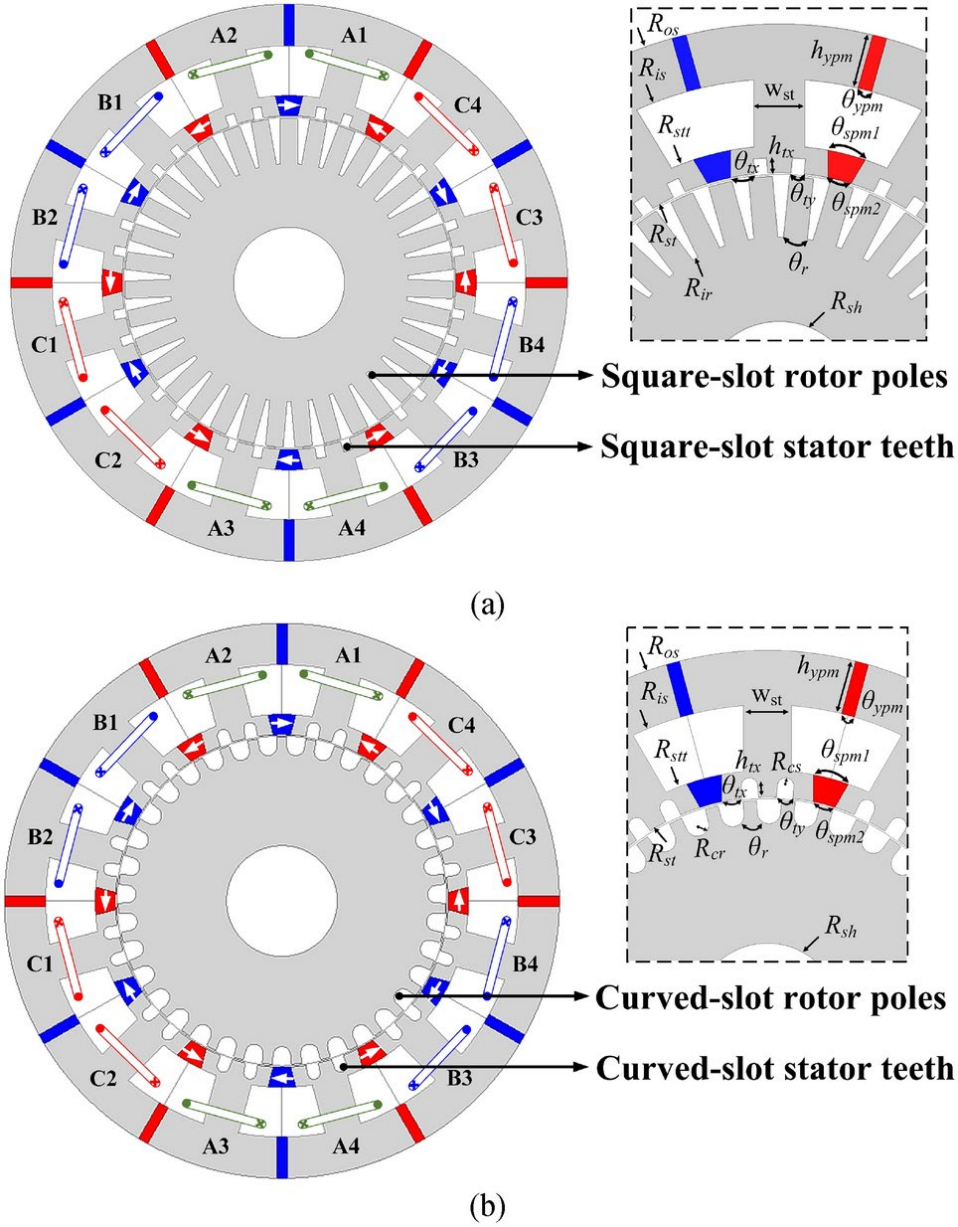
The cross-section topologies with their parameter models of 12/3/35-poles (*k* = 2) of MT-DDSPM: (**a**) square and (**b**) curved slots.

The selected 12/3/35-poles (stator poles/stator teeth/rotor poles-pole) structures of the MT-DDSPM with conventional square and the proposed curved slots are presented in Fig. 1a and Fig. 1b, respectively. These structures consist of a three-phase armature coil installed at the stator slot, big salient stator poles split into multi-tooth to improve torque production because of an arranged magnetic flux path, and lightweight salient rotors. A dual-PM source mounted at the slot opening and stator yoke produces a combined magnetic flux like that introduction^21^. The number of PMs is twice the number of stator poles. Moreover, the capability of their doubly salient pole provides low-velocity operation, which can be used in many applications, such as pushback tracks and industrial pumps.

**Fig. 2:**
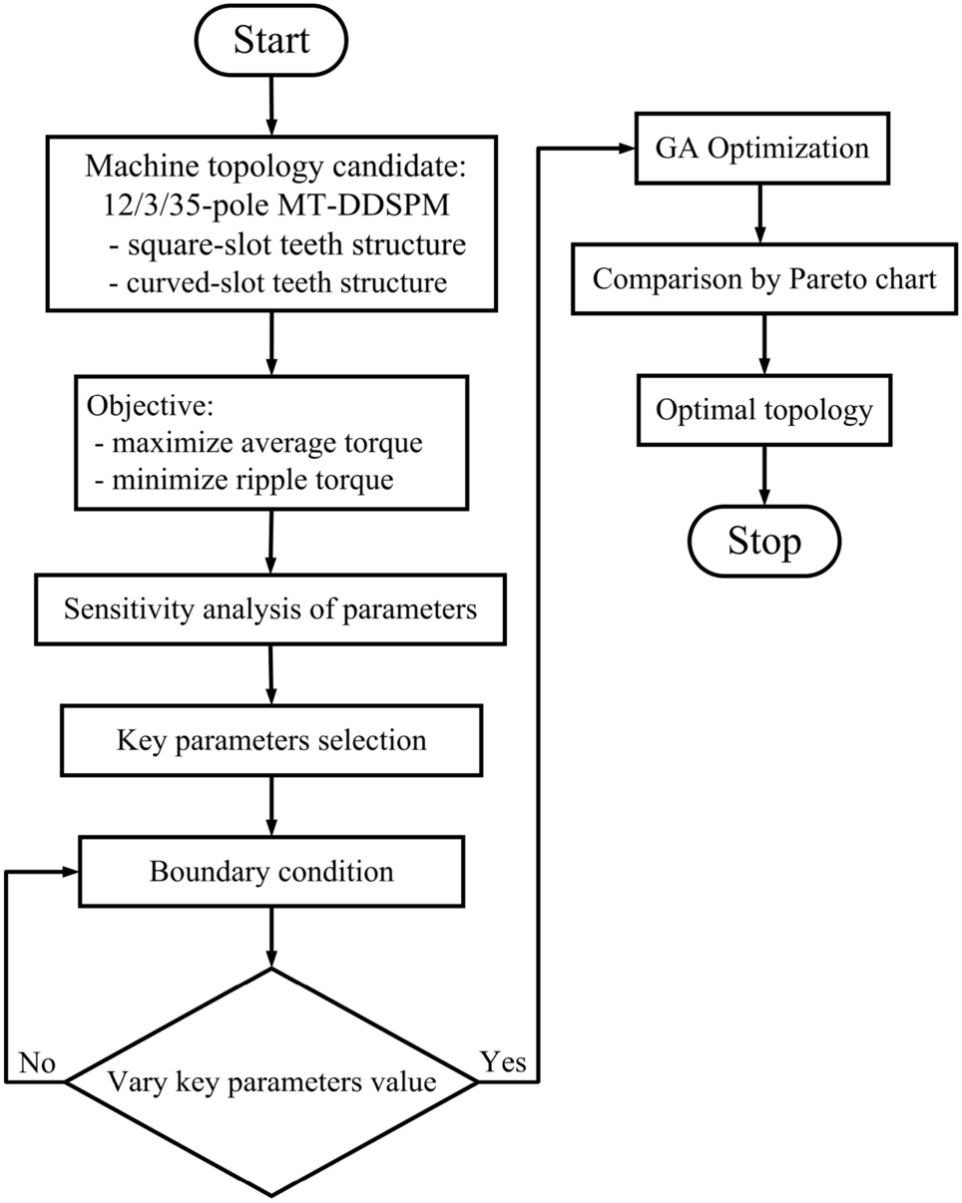
Optimization process.

The characteristic of curved slots is that most parts of the structures are similar to that of the square slots except for the slot characteristics of spitted stator teeth and rotor poles. These curved slots are characterized by the curvature design, which significantly encourages a more uniform and smooth magnetic flux path, focusing only on the edge and slot, to improve the magnetic flux concentration while flowing in the stator teeth. Then, the radius of the curved slots is a significant factor of the proposed structure, which is called the curvature radii. It can be separated into two parts depending on their locations, which are the curved-slot stator teeth and the curved-slot rotor poles. The curved-slot stator-teeth radius, *R*_*cs*_, is achieved by

2 $$R_{cs} = \frac{{\pi R_{st} \theta_{ty} }}{360}$$

where *R*_*st*_ is the inner stator radius and *θ*_*ty*_ is the stator tooth slot arc. As indicated in the parameter models of Fig. 2, the varying *θ*_*ty*_ influences the *θ*_*tx*_ acting as a determinator of the quantity of the magnetic flux passing through stator teeth.

The curved-slot rotor-pole radius, *R*_*cr*_, is achieved by

3 $$\left\{ \begin{gathered} \theta_{cr} = \frac{1}{{2N_{r} }}\left[ {360 - \frac{{(R_{st} - g)N_{r} \theta_{r} }}{{R_{ir} }}} \right] \hfill \\ R_{cr} = \frac{{\pi R_{ir} \theta_{cr} }}{180} \hfill \\ \end{gathered} \right.$$

where *g* is the air-gap length, *θ*_*r*_ is the rotor pole arc and *R*_*ir*_ is the inner rotor radius. The *θ*_*r*_ and *R*_*ir*_ are the determinators of the distance of the magnetic flux circulation.

It can be seen that the *R*_*cs*_ and *R*_*cr*_ play a crucial role in the magnetic flux path and slot shape around the stator and rotor teeth of the structure. So, the parameters of the curved slots will be optimized to aim for high torque potential production.

### Optimization design

Figure 2 presents an overall optimization process. The 12/3/35-pole MT-DDSPM with the square and curved slots is optimized to reach suitable parameters. The objective is to achieve the best torque potential production: high average and low ripple torque. The analytical sensitivity index of the key parameters that affect torque potential production is calculated, as shown in Table 3. The *θ*_*ty*_ and *θ*_*r*_ have values close to 1, indicating more influence on torque potential when compared with the *R*_*ir*_^36^. The key parameters, including *θ*_*ty*_, *θ*_*r*_ and *R*_*ir*_ are chosen for consideration in this optimization. The *θ*_*ty*_ affects the quantity of the magnetic flux at stator teeth for flux linkage induction. The *θ*_*r*_ impacts the magnetic flux movement passing through an air gap. *R*_*ir*_ determines the distance of the magnetic flux circulation. Moreover, these selected key parameters of the machine relate to the *R*_*cs*_ and *R*_*cr*_ for the curved-slot structure. Then, the boundary conditions of the parameters determine no lower and greater than 20 percent of the default parameter while attempting to keep the armature slot and PM consistent. This condition is established because the variation in over-size *θ*_*ty*_ and *θ*_*r*_ is acquired with the reduction in flux quantity and structural robustness. The genetic algorithm (GA) is used to verify global optimization because of its ability to explore a wider solution space and avoid local optima. The 30 generations of random individuals and 100 populations of each generation are assigned. The crossover probability is about 0.9 and the mutation probability is about 0.1 in the process of the reproduction setup. The optimization results of this machine are stored and compared in terms of a Pareto chart. The optimal structure of this machine is selected from the Pareto chart using the function of the trade-off evaluation^37^.

**Table 3:** Boundary conditions of the parameter selection.

Parameter	Unit	Sensitivity index	Value	
			Min	Max
Stator tooth slot arc, *θ*_*ty*_	degree	0.60	2	5
Rotor pole arc, *θ*_*r*_	degree	0.75	2	7
Inner rotor radius, *R*_*ir*_	mm	0.36	52	70

**Fig. 3:**
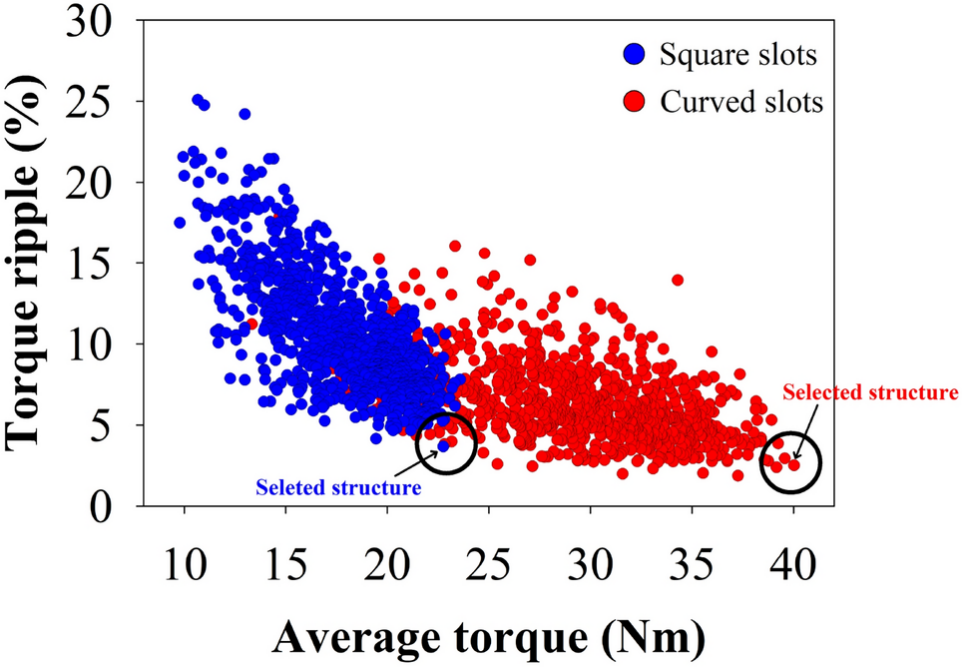
Optimization results in Pareto chart.

Figure 3 illustrates the Pareto chart, which provides the average versus ripple torque of the different selected parameters for the possible 12/3/35-pole MT-DDSPM with square (blue point) and curved slots (red point). The optimal point was selected based on the defined optimization objective, which is maximizing average torque and minimizing ripple torque below 2%. Furthermore, some point increase in over torque led to diminishing returns and structural instability. So, the MT-DDSPM structures, indicated by the black arrow, are chosen for their ability to produce high average torque over a wide range while minimizing ripple through their well-suited parameters. As a result, the optimized *θ*_*ty*_, *θ*_*r*_ and *R*_*ir*_ obtained by the trade-off evaluation function in the Pareto chart as well as other parameters of the square- and curved-slot MT-DDSPM are given in Table 4.

**Table 4:** Design specifications of the MT-DDSPM with square and curved Slots.

Parameter	Unit	Optimal structure	
		Square slots	Curved slots
Stator pole number, *N*_*s*_	poles	12	
Stator teeth number,* n*	poles	3	
Rotor pole number,* N*_*r*_	poles	35	
Rated speed	rpm	1000	
Axial length, *l*	mm	125	
Air-gap length, g	mm	1.25	
Number of turns/pole	turns	68	
Outer stator radius, *R*_*os*_	mm	125	
Inner stator yoke radius, *R*_*is*_	mm	106.5	
Inner stator radius, *R*_*st*_	mm	75	
Stator tip radius, *R*_*stt*_	mm	84	
Spilt ratio		0.59	
Inner rotor radius, *R*_*ir*_	mm	53.6	69.74
Shaft radius, *R*_*sh*_	mm	25	
Stator tooth height, *h*_*tx*_	mm	5	
Stator pole width, *w*_*st*_	mm	16.33	
Stator tooth arc, *θ*_*tx*_	degree	5.87	5.12
Stator tooth slot arc, *θ*_*ty*_	degree	3.26	4.39
Rotor pole arc, *θ*_*r*_	degree	6	3.5
Curved-slot stator-teeth radius, *R*_*cs*_	degree	–	2.87
Curved-slot rotor-pole radius, *R*_*cr*_	degree	–	4
Yoke PM height,* h*_*ypm*_	mm	18.5	
Yoke PM arc, *θ*_*ypm*_	degree	2.1	
Slot PM arc 1, *θ*_*spm1*_	degree	8.47	
Slot PM arc 2, *θ*_*spm2*_	degree	5.85	
PM material		NdFeB-35	

**Fig. 4:**
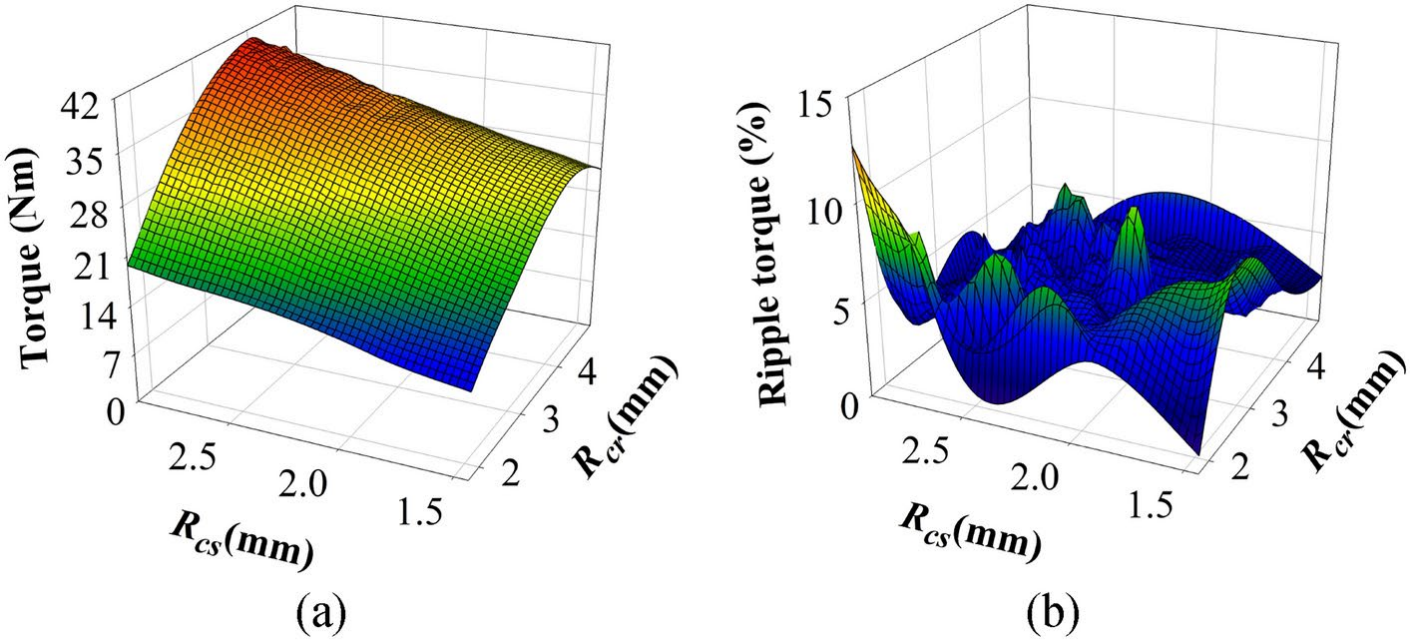
Influence of the curved slots on (**a**) rated torque and (**b**) ripple torque.

Additionally, the whole parameter results of the curved-slot MT-DDSPM are calculated to analyze the curvature radii, consisting of the *R*_*cs*_ and *R*_*cr*_ based on (2) and (3), respectively. Figure 4a indicates the rated torque versus the relation between *R*_*cs*_ and *R*_*cr*_. It is seen that the rated torque increases when the *R*_*cs*_ are increased with expanding *θ*_*ty*_ since the area of stator teeth is reduced, affecting a more magnetic flux concentration while the quantity remains. However, the over-width of *R*_*cs*_ results in a fragile machine structure. Meanwhile, the rated torque is increased with the *R*_*cr*_ increases from 2 to 3 mm due to the *θ*_*r*_ expansion and *R*_*ir*_ reduction, and then decreased with the *R*_*cr*_ increases over 4 mm. This is explained by the impact of the large leakage flux occurrence when the *R*_*cr*_ has an excessive width as well as *θ*_*r*_ is too thin. The ripple torque with variation of *R*_*cs*_ and *R*_*cr*_ is shown in Fig. 4b. The inconsistent ripple torque waveform is obtained when varying the *R*_*cs*_ and *R*_*cr*_ due to the asymmetrical magnetic flux from their stator/rotor poles. The ripple torque lower than 5% shows a good diameter on the *R*_*cs*_ and *R*_*cr*_ for this curved-slot structure.

Consequently, the analysis of the curvature radii found that the *R*_*cs*_ of 2.87 mm and *R*_*cr*_ of 4.00 mm obtained from the optimal selected parameters as mentioned above, are the suitable curvature radii of curved slots in MT-DDSPM with high-rated torque and low ripple torque over the other one in this work. Then, the electromagnetic performance of both MT-DDSPM structures will be deeply analyzed with compared for fair constraint.

## Analysis of simulation results

The comparative electromagnetic performance between the square and curved slots of the 12/3/35-pole MT-DDSPM was investigated by the 2-D FEM. These consist of MEC, magnetic flux distribution, air-gap flux density, MFCI, flux linkage, back-EMF, cogging torque, rated torque, ripple torque and PM demagnetization to verify under no-load, load, and overload constraints. Overall performance was concluded and compared to the initial and traditional structure in fair condition.

### MEC and magnetic flux distribution

**Fig. 5:**
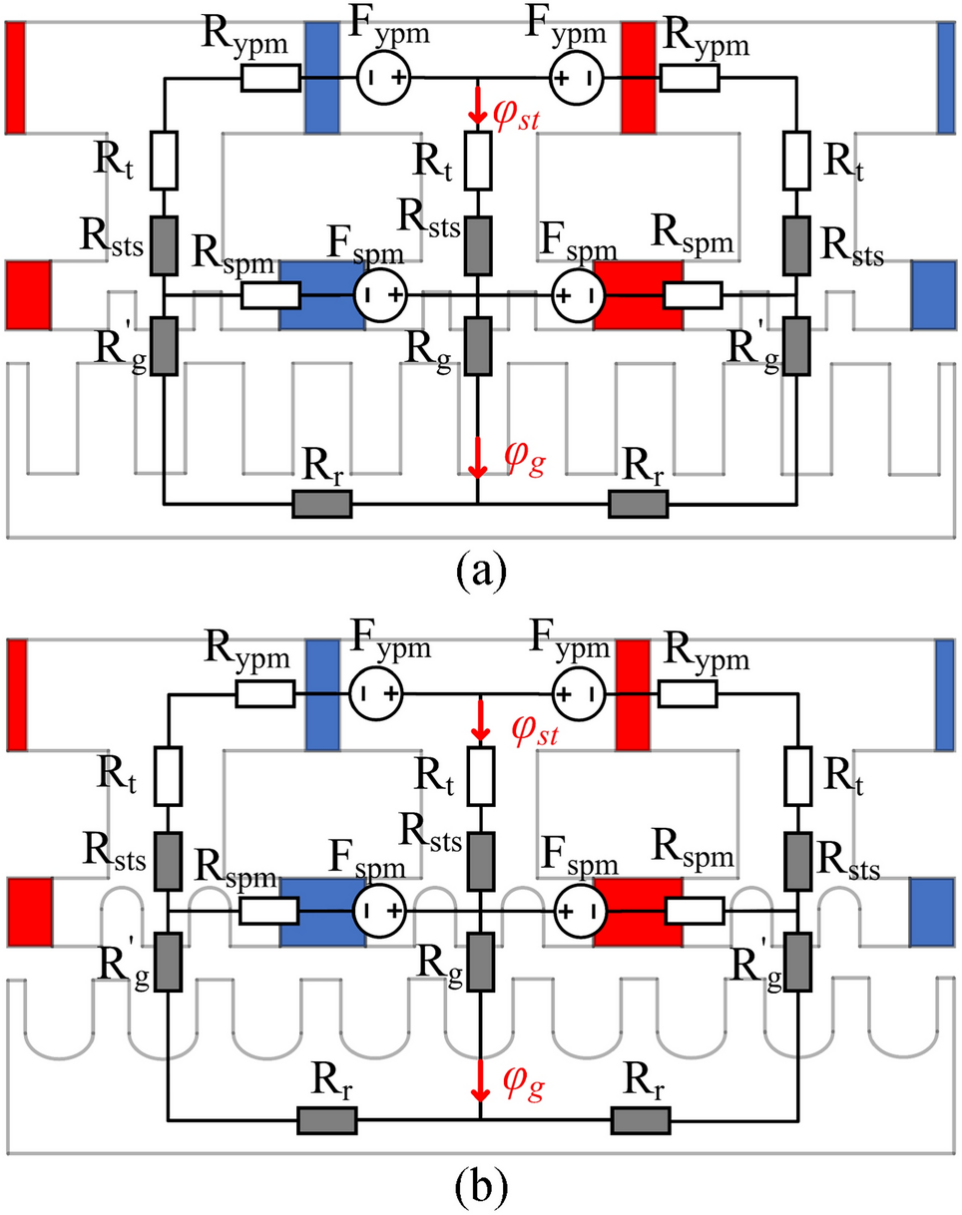
MECs of the 12/3/35-pole MT-DDSPM with (**a**) square and (**b**) curved slots.

The MEC was studied to further explain the difference in the magnetic flux path for the square- and curved-slot MT-DDSPM. Figure 5 shows the MEC of both structures. They exhibit a similar circuit, which means the air-gap flux density, *φ*_*g*_, and flux density at the stator part, *φ*_*st*_, can be calculated in the same equation as

4 $$\varphi_{g} = \frac{{2F_{ypm} }}{{R_{stator} + R_{gap} }} + \frac{{R_{stator} }}{{R_{p} }}F_{spm}$$

5 $$\varphi_{st} = \frac{{2F_{ypm} }}{{R_{stator} + R_{gap} }} - \frac{{2R_{gap} }}{{R_{p} }}(F_{ypm} - F_{spm} )$$

where *F*_*ypm*_ and *F*_*spm*_ is the magnetomotive force of the yoke PM and slot PM. the whole reluctance, including *R*_*stator*_, *R*_*gap*_ and *R*_*p*_, can be obtained as

6 $$\left\{ \begin{gathered} R_{stator} = R_{ypm} + R_{t} + R_{sts} \hfill \\ R_{gap} = R_{rotor} + 2R_{g} + R_{g}{\prime} \hfill \\ R_{p} = 2R_{stator} R_{spm} + 2R_{gap} (R_{stator} + R_{spm} ) \hfill \\ \end{gathered} \right._{{}}^{{}} ;_{{}}^{{}} R = \frac{l}{{\mu_{r} \mu_{0} A}}$$

The reluctance is calculated from the relation of the length and the area at the magnetic flux path. The change in the reluctance affects the air-gap magnetic flux, the quantity of the magnetic flux at stator teeth, and the distance of the magnetic flux circulation, resulting in the magnetic flux concentration, afterward. However, the MT-DDSPM is added by curvature radii, the *R*_*sts*_, *R*_*r*_, and *R*_*g,*_ values have differences from the square-slot structure, affecting the variation of the *φ*_*g*_ and *φ*_*st*_.

**Fig. 6:**
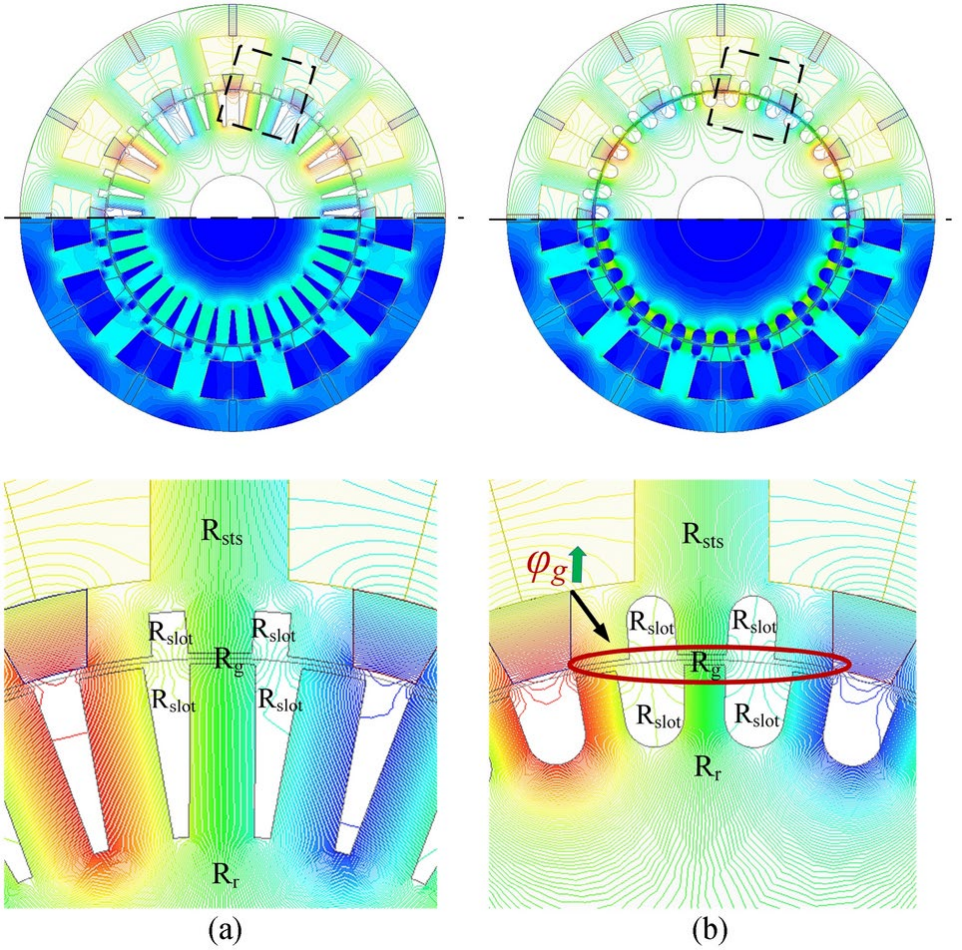
No-load magnetic flux distribution and their magnetic flux paths of the MT-DDSPM: (**a**) square and (**b**) curved slots.

Figure 6 shows the no-load magnetic flux distribution of both machine structures. The magnetic flux passes through the air gap and connects the multi-stator teeth and rotor^21^. Focusing on the stator pole part, the added *R*_*cs*_ in the curved-slots MT-DDSPM indicate an increase of reluctance at the slots, *R*_*slot*_, which the area of stator teeth slot is wider than the square-slots one, and then *R*_*sts*_ is reduced due to the area reduction of the stator teeth. This improves concentration and remains the quantity of the magnetic flux as mentioned in the previous section. In the rotor pole part, the added *R*_*cr*_ in the MT-DDSPM also indicates an increased *R*_*slot*_, resulting in *R*_*r*_ reduction with the enhancement of the magnetic flux concentration. Moreover, the *R*_*ir*_ of the curved slots is larger than that of the square slots. This decreases the distance of the magnetic flux circulation thereby further reducing *R*_*r*_. Especially, the variation of the *R*_*sts*_ and *R*_*r*_ has a continuous impact on the *R*_*g*_.

Normally, the solution of the *φ*_*g*_ and *φ*_*st*_ has no account for the *R*_*slot*_ as it is not part of the magnetic flux path. However, the *R*_*slot*_ is only utilized to explain *R*_*sts*_, *R*_*r*_, and *R*_*g*_ variation. So, the *φ*_*g*_ and *φ*_*st*_ in the curved-slot MT-DDSPM are higher than that of the square-slot MT-DDSPM because of the reluctance value reduction of *R*_*sts*_, *R*_*r*_, and *R*_*g*_. These reasons will be utilized to support the description in the following section.

### Air-gap flux density

**Fig. 7:**
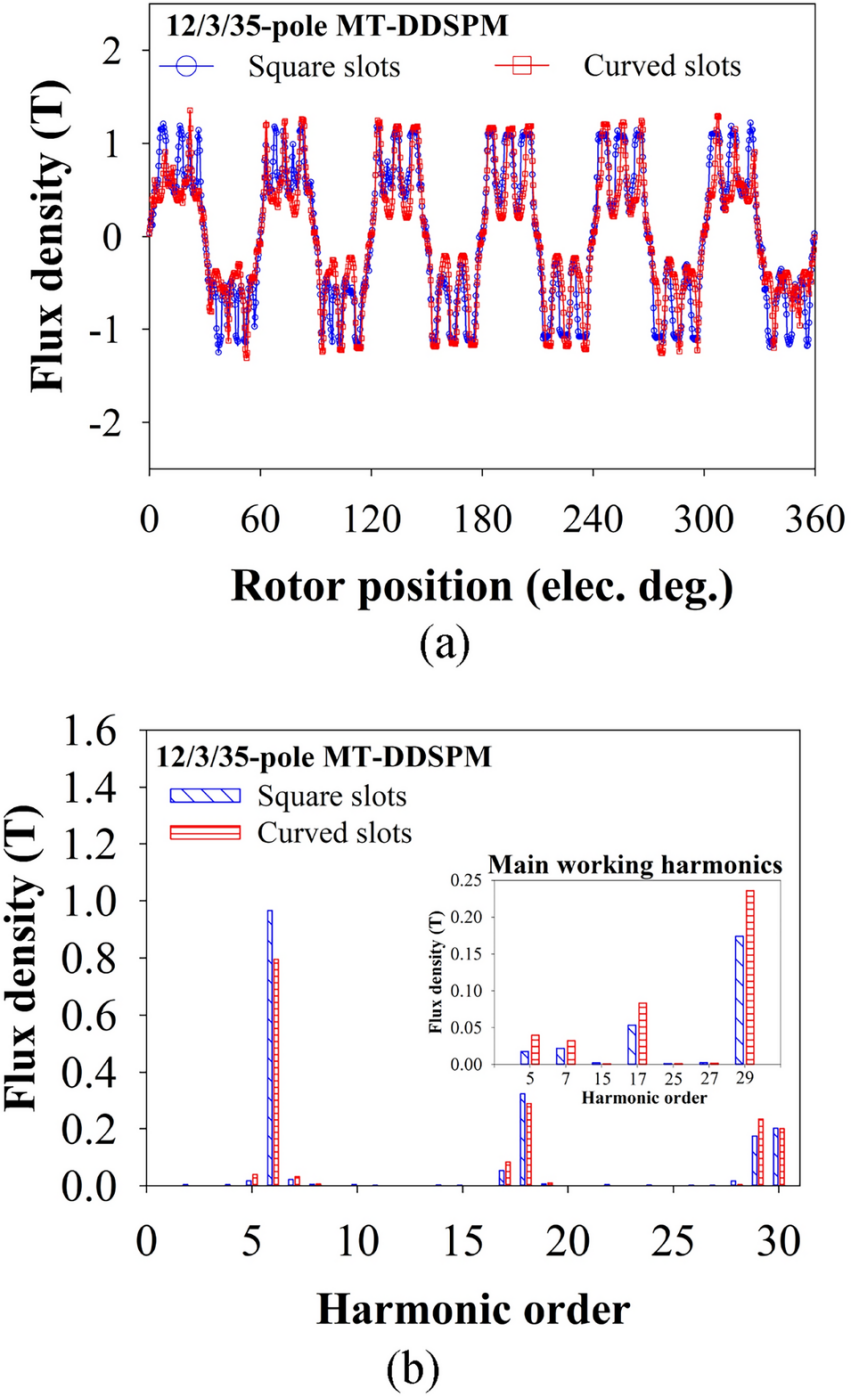
Air-gap magnetic flux density of the 12/3/35-pole MT-DDSPM: (**a**) waveform and (**b**) whole harmonic spectra with main working harmonic.

Figure 7a indicates the air-gap flux density of the optimized MT-DDSPM with the curved slots compared to the square slots. The total air-gap flux density of the curved slots is slightly higher in amplitude and is similar to waveform characteristics. This is the MEC result of its *φ*_*g*_ increment. The flux density harmonics of those structures were analyzed, shown in Fig. 7b. The principal pole-pair number of the multi-tooth double salient family is |(*N*_*s*_/2)*k* ± *N*_*r*_|, as discussed in^38^. The main working harmonics related to the characteristics of the aira-gap flux density were selected, as concluded in a subfigure of Fig. 7b. They consist of 5th (|5(*N*_*s*_/2) ˗ *N*_*r*_|), 7th (|7(*N*_*s*_/2) ˗ *N*_*r*_|), 17th (|3(*N*_*s*_/2) ˗ *N*_*r*_|) and 29th (|(*N*_*s*_/2) ˗ *N*_*r*_|). It is seen that the main working harmonics of the curved slots are higher than those of the square one, especially harmonic numbers of 5th, 7th, 17th and 29th. These harmonics will have an important impact on subsequent torque production.

### Magnetic flux concentration indicator

Several methods exist to quantify magnetic flux concentration in electrical machines, such as harmonic flux density observation, analysis of the lumped parameter models and magnetic field visualization. These methods focus on the overall behavior of the magnetic flux distribution rather than magnetic flux concentration. The MFCI is invented in this work to specifically quantify how magnetic flux is concentrated at critical machine regions.

**Fig. 8:**
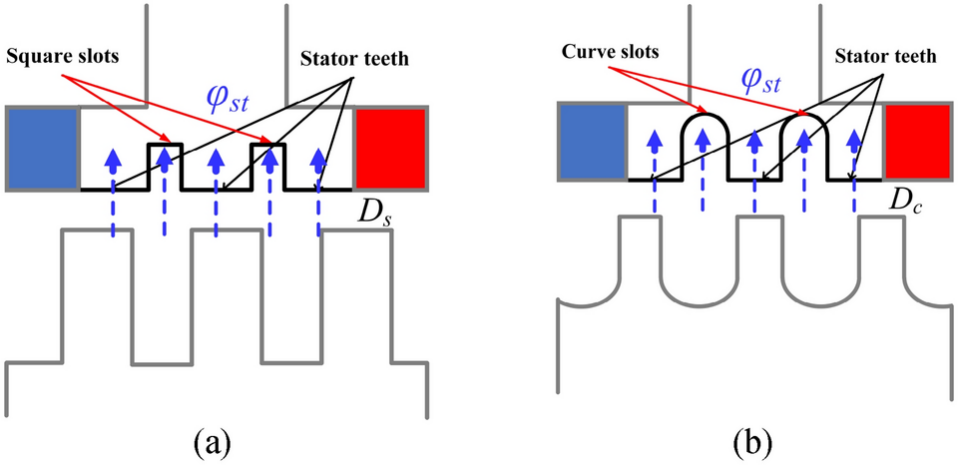
Positions for MFCI consideration of the MT-DDSPM: (**a**) square and (**b**) curved slots.

Figure 8 shows the position of the MFCI consideration because this region represents magnetic flux consideration while moving through the stator teeth for processing the flux linkage induction at the winding. This is focused only on the stator teeth and stator teeth slot of one pole, which can be obtained by

7 $$\theta_{N} = \frac{{180(D_{s,c} )}}{{\pi R_{st} }}\left\{ \begin{gathered} D_{s} = \underbrace {{\left[ {2nR_{st} \theta_{tx} + R_{st} \theta_{spm2} } \right]\frac{\pi }{180}}}_{Teeth} + \underbrace {{\left[ {\frac{{R_{st} \theta_{ty} \pi }}{90} + 4h_{tx} } \right]}}_{Slot} \hfill \\ D_{c} = \underbrace {{\left[ {2nR_{st} \theta_{tx} + R_{st} \theta_{spm2} } \right]\frac{\pi }{180}}}_{Teeth} + \underbrace {{\left[ {2R_{cs} \pi + 4h_{tx} } \right]}}_{Slot} \hfill \\ \end{gathered} \right.$$

where *θ*_*N*_ is the considered position of one pole,* D*_*s*_ and *D*_*c*_ are the distances considered of square and curved slots, respectively.

**Fig. 9:**
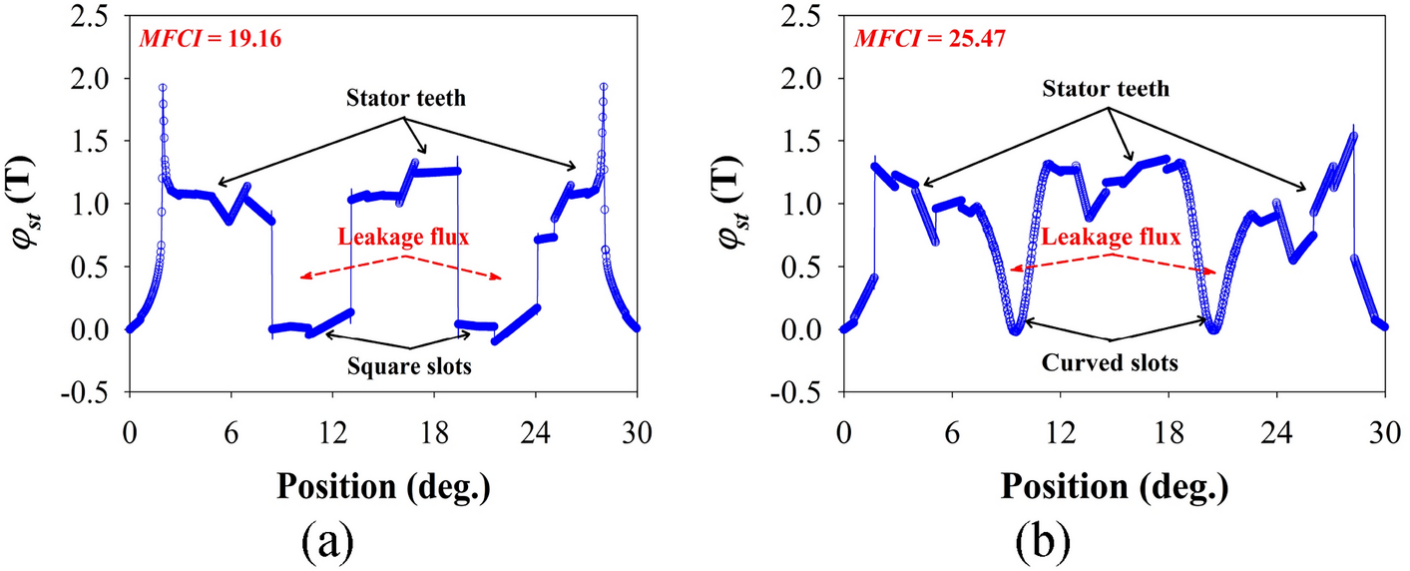
The magnetic flux density at the stator teeth of the 12/3/35-pole MT-DDSPM with (**a**) square and (**b**) curved slots.

The *φ*_*st*_ of the square- and curved-slot MT-DDSPM is investigated by varying *θ*_*N*_ as shown in Fig. 9. The waveform characteristics look like their structures, including the stator teeth and stator teeth slots. The stator teeth region indicates the quantity of the magnetic flux density distributing into stator teeth. The curved slots have more stability of *φ*_*st*_ of about 1.3 T than the square slots, indicating better magnetic loading conditions. Moreover, the *φ*_*st*_ obtained by the square slots is over 2.0 T. This results in a big problem in the form of heat and loss for long-term operation. Focusing on the feature of stator teeth slot for both structures show different forms according to their characteristic structures. The amount of the leakage flux is based on the slot area volume. It can be observed that the curved slots produce a lower leakage flux with a smaller area volume than the square slots due to a more uniform and smooth magnetic flux path.

The MFCI is created by the formula of the absolute surface roughness and applied to study in terms of magnetic flux concentration. It can be calculated from *φ*_*st*_ at each position as

8 $$MFCI = \frac{1}{{N^{2} }}\sum\nolimits_{i = 1,2,3,...}^{N} {\left| {\varphi_{st} (\theta_{i} )} \right|}$$

where *N* is the number of steps for consideration.

The MFCI obtained by curved slots is 25.47 compared to 19.16 obtained by square one, which is 32.93% higher. This means that the curved slots can receive better magnetic flux concentration at stator teeth with more stability and lower leakage flux. This can improve the quantity of magnetic flux for the flux linkage induction and the other electromagnetic characteristics of the machine.

### Flux linkage and back-EMF

**Fig. 10:**
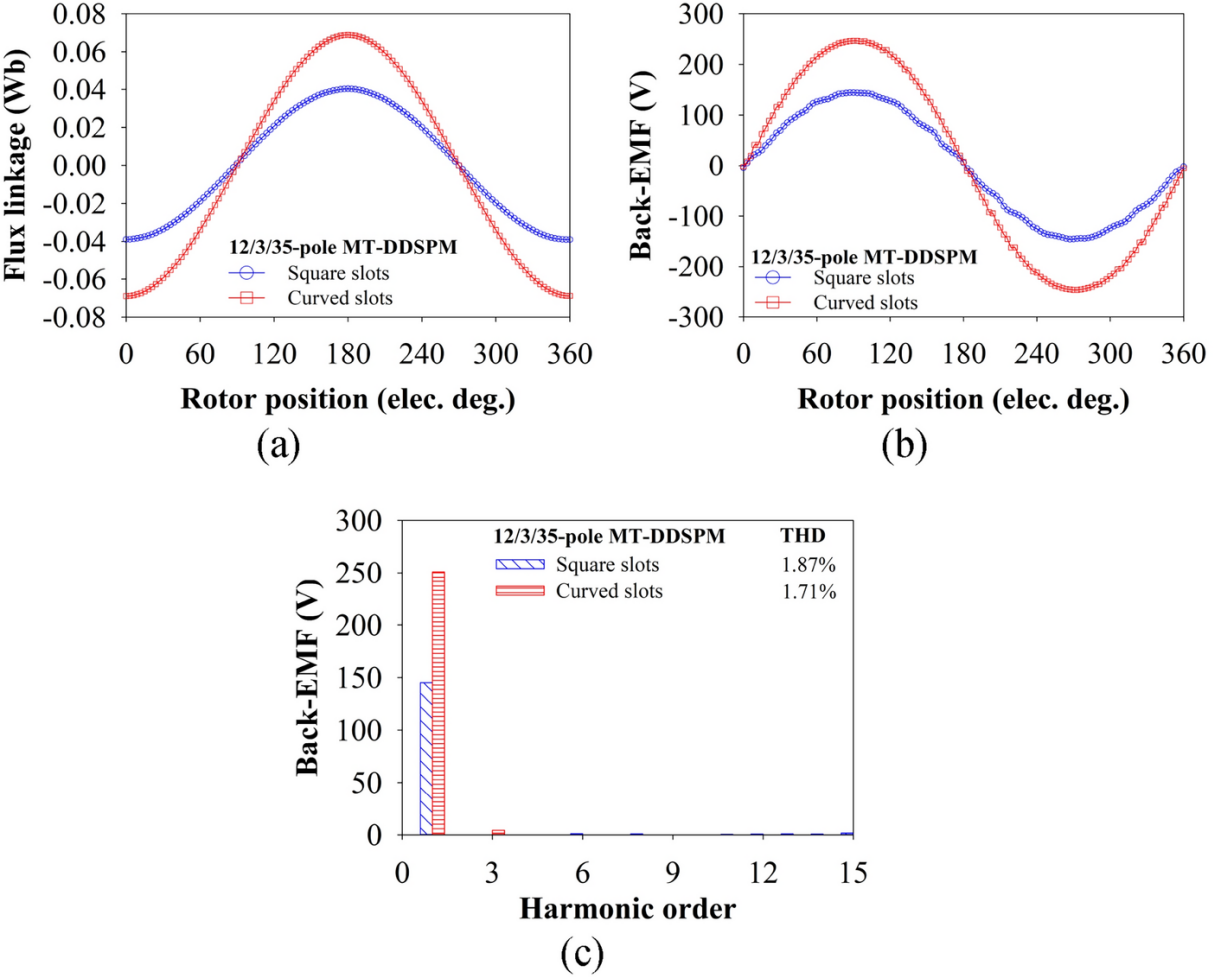
Comparative results of the 12/3/35-pole MT-DDSPM: (a) flux linkage, (**b**) back-EMF waveform and (**c**) back-EMF harmonic at 1000 rpm.

In Fig. 10a, the flux linkage of the MT-DDSPM is studied with varying rotor positions. The flux linkage produced by the curved slots has a higher magnitude than that produced by the square slots because of the higher MFCI with improved magnetic flux concentration at the stator teeth as discussed in the previous section. This reason also results in the sinusoidal back-EMF of the curved slots, which is about 70.76% larger when compared to square one, as illustrated in Fig. 10b. In addition, both structures are categorized into a low range of THD compared with the other doubly salient structures in this work because there is a minor disturbance from the flux leakage, shown in Fig. 10c.

### Torque characteristics

The torque characteristics are validated in this section, including the cogging torque, rated torque and ripple torque under fixed load and over-load condition.

**Fig. 11:**
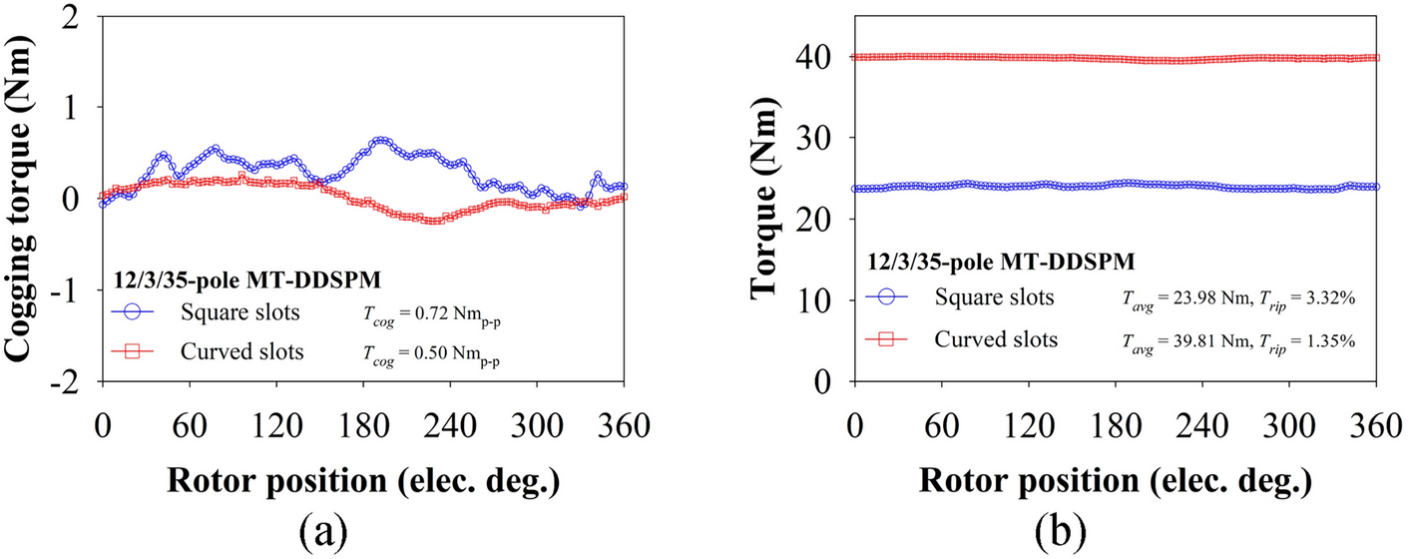
Comparative torque characteristics of 12/3/35-pole MT-DDSPM: (**a**) cogging torque and (**b**) rated torque.

Figure 11a illustrates the cogging torque of the 12/3/35-pole MT-DDSPM with various rotor positions. It can be seen that the cogging torque of both structures shows an asymmetric waveform because the selected stator/rotor pole suffers significantly from an unbalanced magnetic flux. The cogging torque is the normal magnetic flux tension between the stator teeth and the rotor poles. These curved-slot MT-DDSPM can generate a smaller peck-to-peak cogging torque of about 0.51 Nm_p-p_ compared to the square MT-DDSPM, which generates around 0.72 Nm_p-p_. This is because the added *R*_*cs*_ and *R*_*cr*_ help to expand the area of the stator teeth slot and then the *θ*_*tx*_ and *θ*_*r*_ reduction, decreasing the tension of the stator teeth and the rotor poles, afterward. However, machine structures show a low range of the cogging torque and an easy starting operation.

The rated torque of the proposed MT-DDSPM is compared at a fixed rated current of 8 A and *J*_*load*_ of 1.4 A/mm^2^^30^. As shown in Fig. 11b, the curved slots can provide 39.81 Nm of the rated torque twice higher than the square one because the back-EMF and cogging torque profiles are obtained from the enhanced magnetic flux concentration using curvature-shape slots. Moreover, the lowest ripple torque is about 1.35% found at the curved-slot teeth because of low peck-to-peak cogging torque and THD of back-EMF. This affects the decrease of the acoustic noise from machine vibration at operation time.

**Fig. 12:**
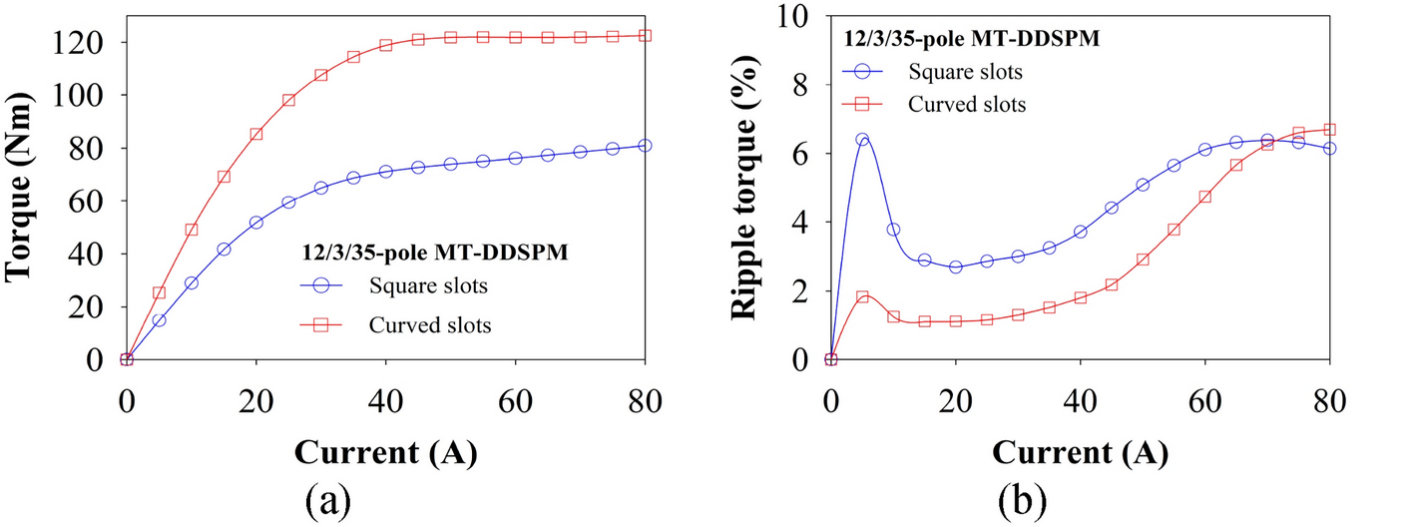
Comparative results of 12/3/35-pole MT-DDSPM: (**a**) cogging torque and (**b**) rated torque.

The over-load current of the proposed MT-DDSPM is studied as presented in Fig. 12. At the high load current, both square and curved slots show similar characteristics because of the stability of the magnetic flux in their structures, shown in Fig. 12a. However, the curved slots provide a higher torque profile than square slots due to an improvement in the magnetic flux concentration. Figure 12b indicates the exact behavior of the ripple torque for those machine structures. They illustrate the high ripple torque percentile when assigned the current of 5 A. This reason explains the vibration that occurred from the attentive force of the magnet and rotor while the machine was starting. After increasing more than 5 A, the ripple torque suddenly drops and slightly increases until 40 A, indicating a steady state. Then, the increased load current from 50 to 60 A results in a higher ripple torque because the over-saturated magnetic flux with leakage occurs, affecting higher temperature and increased machine vibration. At the highest current ranges from 70 to 80 A, the ripple torque of the curved slots and the square one indicates the maximum limit of thermal and vibration that the machine cannot handle before stopping operation. So, the best load current range for these machines should be between 8 to 40 A at steady-state operation, which ensures the machine operates safely. The analytical results show that the MT-DDSPM with the curved slots has better torque production at over-load capability.

### PM demagnetization

**Fig. 13:**
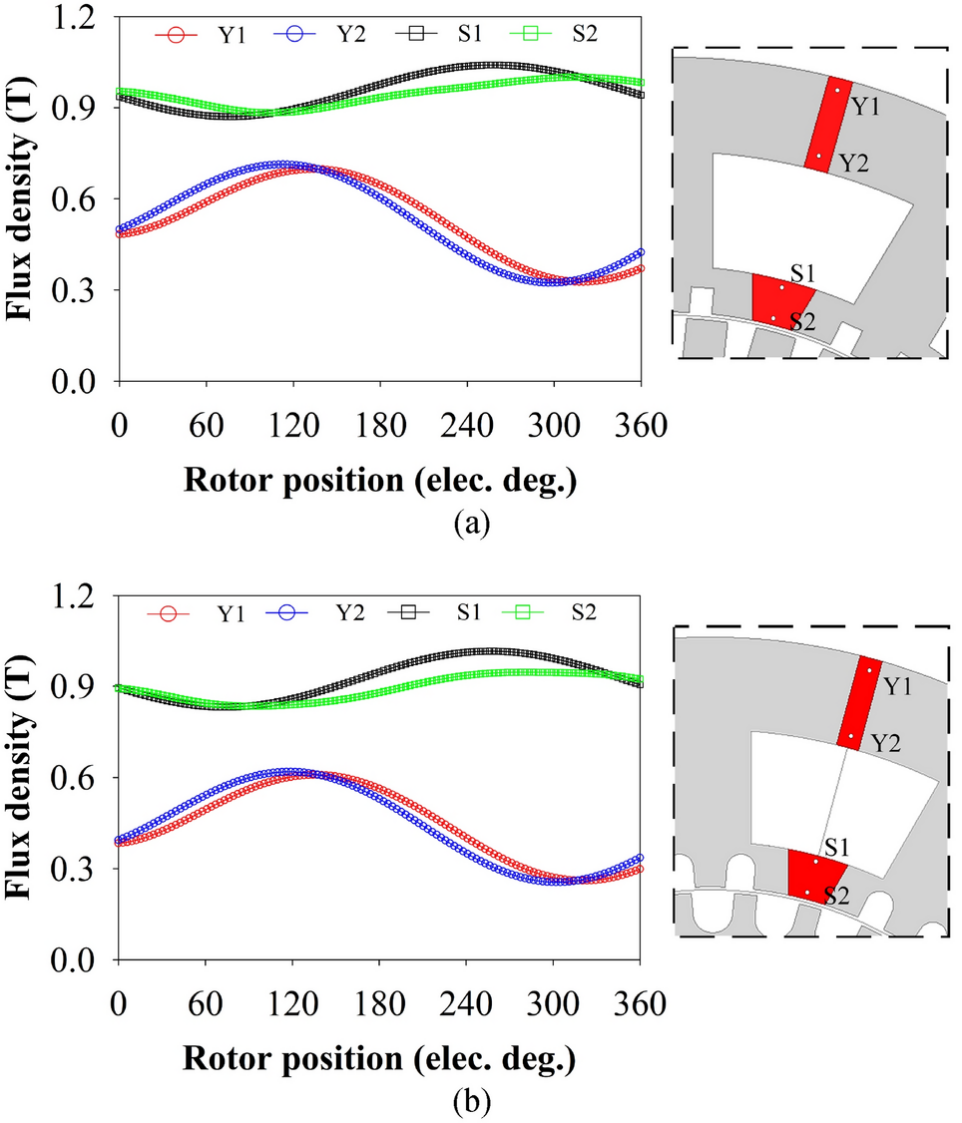
Demagnetization analysis of 12/3/35-pole MT-DDSPM in each simple point: (**a**) square and (**b**) curved slots.

The PM demagnetization risk of the 12/3/35-pole MT-DDSPM with square and curved slots was studied at the rated current of 8 A and temperature of 100 °C. The flux density profiles of the yoke PM and slot PM made from NdFeB-35 material were analyzed in each sample point of both proposed structures, as shown in Fig. 13. The waveform helps to explain the range of the demagnetization risk, with the maximum point representing the critical threshold and the minimum point representing knee demagnetization point^39^. The critical threshold point for the NdFeB-35 material is about 1.1 T. When the flux density exceeds this critical threshold, the machine structure suffers high magnetic loading. Meanwhile, the knee demagnetization point for the NdFeB-35 material is about 0.1 T. When flux density falls below the knee demagnetization point, the machine structure suffers a high risk of demagnetization. The results show that the flux density of the curved slots at Y1 and Y2 has values that reached knee demagnetization points when compared with that of the square slot due to the most magnetic flux of the curved slot crowned at stator teeth. Additionally, the flux density at S1 and S2 obtained by both structures is a quite high value close to a critical threshold. When increasing the temperature over 100 °C, the machine structure may be more at risk of magnetic loading. However, the PM demagnetization risk in the curved-slot MT-DDSPM is found to be insignificantly different compared to the square slots, which fall within a low level and can be operated safely.

### Comparative study

A comparative study in terms of the overall performance of the machines under the same diameter is given in Table 5. It exhibits that the torque characteristic of the optimized 12/3/35-pole MT-DDSPM structure is improved using curved slots compared to square slots even in the initial and traditional structure. It is seen that the MT-DDSPM utilized by curved slots increases efficiency by about 5% more than the other machines under similar amplitude of losses. Especially, the highest MFCI obtained by this machine is presented compared to other machines. This is because of the better quality of the magnetic flux concentration using curved slots. As a result, it can prove that the utilization of the curvature-shape slots is involved in the overall performance of the electrical machine.

**Table 5:** Comparative study of the 12/3/35 pole MT-DDSPM.

Item	Traditional structure	Initial structure		Optimized structures	
	12/13 pole^21^	6/2/13 pole^25^	12/3/35 pole	12/3/35 pole square-slot teeth	12/3/35 pole curved-slot teeth
Maximum back-EMF (V)	101.12	118.12	121.04	124.69	249.24
Cogging torque (Nm_p-p_)	3.4	2.60	0.52	0.73	0.51
Average torque (Nm)	15.3	19.47	19.95	23.98	39.81
Ripple torque (%)	27.19	13.9	2.65	3.32	1.35
Copper loss (W)	68.75	–	35.80	40.59	40.58
PM loss (W)	–	–	16.61	17.44	15.75
Core loss (W)	–	–	15.30	21.74	19.89
Efficiency (%)	–	–	90.9	90.85	94.97
PM volume (mm^3^)	3.57 × 10^5^	4.69 × 10^5^	3.33 × 10^5^	2.62 × 10^5^	2.62 × 10^5^
MFCI	16.13	11.90	17.94	19.16	25.47

The strengths and limitations of the curvature-shape slots are compared with other state-of-the-art designs in this literature. Novel machine designs are proposed annually as presented in Table 6. These techniques are currently very popular. For example, the Halbach PM array design, proposed in 2007, has gained attention for its benefits. This design is quite in trend for use with electric vehicle motors due to its reduction of magnetic interference. However, the limitation of this design is the hard control of the magnetic direction. In 2023, the multi-tooth DDSPM was introduced as the latest version of the DSPM structure. The new structure can provide an enhancement of the flux distribution, especially, at the air gap. Nevertheless, the larger flux leakage is obtained by the non-concentrated magnetic flux caused by many stator teeth. Based on the comparative result, this study intends to solve this problem of the multi-tooth DDSPM in 2023 by using the curvature-shape slots and analyzing them in depth to identify the optimal design.

**Table 6:** Comparative study of the state-of-the-art design.

Structural design	Novelty	Strength	Limitation
Halbach PM arrays design^40,41^	2007	Reduced magnetic interference	Hard magnetic field direction control
Partitioned-stator design^42^	2014	Improved thermal management	Assembly Complexity
Hybrid-excited slot-PM design^15–17^	2015	Better magnetic flux control	Energy Losses
Biased flux PM design^18,19^	2016	Good air-gap flux interaction	Low air-gap flux density
Dual-PM design^20,21^	2019	Combined magnetic fields	Magnet cost
The latest multi-tooth design^24,26^	2023	Improved air-gap flux distribution	Non-concentrated magnetic flux
Curved slot design	2025	Enhanced magnetic flux concentration	High magnetic loading at the air gap

From the results, it can be summarized that the 12/3/35-pole MT-DDSPM applying the curved slots provides better overall electromagnetic performance than the square slots and other structures in this work because it achieved the highest MFCI of 25.47 is improved by adding the *R*_*cs*_ and *R*_*cr*_. This is positively affected by the better magnetic flux concentration using the curvature slots, resulting in the highest flux linkage. Then, the other performance is also improved including back-EMF, cogging torque, rated torque, and ripple torque. Especially, the rated torque obtained from the curved slots is about 39.28 Nm and 66.01% larger than that obtained from the square slots. Even with the overload current, the curved slots can produce more outstanding results. Also, its demagnetization analysis of both structures shows low levels of risk. Therefore, these are novel alternatives for a technical design and a reliable evaluation of the magnetic flux concentration, which can play an important role in the multi-tooth family of electrical machines.

In the part of experimental validation and future work, this analysis shows strong initial information about the machine performance by using real-world validation, which remains critical. For the next phase of this work, the optimized MT-DDSPM with curved slots will fabricate the physical prototype and be subject to experimental testing. However, this machine structure is complex, assessing the manufacturability and assembly tolerances. Hence, this study seeks to validate the practical feasibility of the proposed machine for industrial applications by combining experimental results with computational analysis.

## Conclusion

This paper innovatively employed the novel curvature-shaped slots in combination with multi-tooth DDSPM to overcome the flux leakage issue and to obtain a superior magnetic flux concentration and distribution. Moreover, the magnetic flux concentration indicator (MFCI) was originally developed to analyze the magnetic flux concentration at stator teeth, providing insight into performance improvement. The structural parameters related to the curved slots in the MT-DDSPM were considered and optimized to obtain the best torque production. The electromagnetic characteristics of the curved-slot MT-DDSPM were investigated by using the 2-D FEM and then verified by the traditional square-slot MT-DDSPM. The results verified that the MT-DDSPM with the optimized curved slots provided an MFCI of 32.93% higher than the square slots, obtaining a better magnetic flux concentration. This indicated a higher flux linkage and generated greater electromagnetic performance, especially the highest-rated torque of 39.28 Nm, which was 66.01% higher than square one. The lowest ripple torque of 1.35% was also provided by the designed curved slots. The curved-slot MT-DDSPM was done with better analytical results at over-load current and low-level risk of PM demagnetization. Therefore, these finding ideas of this paper are a novel curved slot added into the MT-DDSPM for superior performance and another reliable evaluation of the magnetic flux concentration based on MFCI. This approach is the beneficial selector for more development and investigation of multi-tooth electrical machines and their related applications. Although, this analytical simulation indicates the significant improvements in the magnetic flux concentration. Future work will focus on a prototype fabrication and the experimental measurement to confirm this computational prediction.

## Data Availability

The datasets used and/or analysed during the current study available from the corresponding author on reasonable request.

## References

[1] Cheng, M., Hua, W., Zhang, J. & Zhao, W. Overview of stator-permanent magnet brushless machines. *IEEE Trans. Ind. Electron.* **58**(11), 5087–5101 (2011).

[2] Chen, H. Flux-switching permanent magnet machines: A review of opportunities and challenges—Part I: Fundamentals and topologies. *IEEE Trans. Energy Convers.* **35**(2), 684–698 (2019).

[3] Yang, Z., Shang, F., Brown, I. P. & Krishnamurthy, M. Comparative study of interior permanent magnet, induction, and switched reluctance motor drives for EV and HEV applications. *IEEE Trans. Transp. Electrif.* **1**(3), 245–254 (2015).

[4] Vlachou, V. I. et al. Overview on Permanent Magnet Motor Trends and Developments. *Energies* **17**(2), 538–585 (2024).

[5] Cai, S., Kirtley, J. L. & Lee, C. H. Critical review of direct-drive electrical machine systems for electric and hybrid electric vehicles. *IEEE Trans. Energy Convers.* **37**(4), 2657–2668 (2022).

[6] Zhao, W. et al. Design and analysis of a new fault-tolerant linear permanent-magnet motor for maglev transportation applications. *IEEE Trans. Appl. Supercond.* **22**(3), 5200204–5200204 (2012).

[7] Taras, P., Li, G. J. & Zhu, Z. Q. Comparative study of fault-tolerant switched-flux permanent-magnet machines. *IEEE Trans. Ind. Electron.* **64**(3), 1939–1948 (2016).

[8] Wei, F., Zhu, Z. Q., Yan, L. & Qi, J. Investigation of stator/rotor pole number combinations and PM numbers in consequent-pole hybrid excited flux reversal machine. *IEEE Trans. Energy Convers.* **37**(3), 2092–2106 (2022).

[9] Liao, Y., Liang, F. & Lipo, T. A. A novel permanent magnet motor with doubly salient structure. *IEEE Trans. Ind. Appl.* **31**(5), 1069–1078 (1995).

[10] Fan, Y., Chau, K. T. & Cheng, M. A new three-phase doubly salient permanent magnet machine for wind power generation. *IEEE Trans. Ind. Appl.* **42**(1), 53–60 (2006).

[11] Liu, C. Emerging electric machines and drives—An overview. *IEEE Trans. Energy Convers.* **33**(4), 2270–2280 (2018).

[12] Xiong, L. et al. “Torque ripple reduction strategy for doubly salient electromagnetic machine based on current given function”, *IEEE*. *J. Emerg. Sel. Top. Power Electron.* **10**(6), 7486–7501 (2022).

[13] Wu, D., Shi, J. T., Zhu, Z. Q. & Liu, X. Electromagnetic performance of novel synchronous machines with permanent magnets in stator yoke. *IEEE Trans. Magn.* **50**(9), 81 (2014).

[14] Cheng, H. et al. Electromagnetic characteristics analysis and torque ripple reduction for doubly salient pm machine. *IEEE Trans. Energy Convers.* **38**(3), 1659–1668 (2023).

[15] Afinowi, I. A. A., Zhu, Z. Q., Guan, Y., Mipo, J. C. & Farah, P. Hybrid-excited doubly salient synchronous machine with permanent magnets between adjacent salient stator poles. *IEEE Trans. Magn.* **51**(10), 1–9 (2015).26203196

[16] Shen, Y., Lu, Q., Shi, T. & Xia, C. Analysis and evaluation of hybrid-excited doubly salient permanent magnet linear machine with DC-biased armature current. *IEEE Trans. Ind. Appl.* **57**(4), 3666–3677 (2021).

[17] Niu, S., Wang, S. & Zhao, X. Overview of stator slot-opening permanent magnet machines. *IEEE Trans. Transp. Electrif.* **9**(1), 782–804 (2022).

[18] Xu, W., He, M. & Ye, C. Novel synchronous machine with permanent magnet in stator yoke. *IEEE Trans. Appl. Supercond.* **26**(7), 1–5 (2016).

[19] Ming, G. et al. Comparative study of biased flux PM machines having different stator core segments and armature winding configurations. *IEEE Trans. Transp. Electrif.* **8**(3), 3379–3389 (2022).

[20] Shen, Y., Zeng, Z., Lu, Q. & Li, Y. Investigation of a modular linear doubly salient machine with dual-PM in primary yoke and slot openings. *IEEE Trans. Magn.* **55**(6), 1–6 (2019).

[21] Meng, Y., Fang, S., Pan, Z. & Qin, L. A new hybrid-excited doubly salient dual-PM machine with DC-biased sinusoidal current. *IEEE Trans. Appl. Supercond.* **31**(8), 1–5 (2021).

[22] Chen, J. T., Zhu, Z. Q. & Howe, D. Stator and rotor pole combinations for multi-tooth flux-switching permanent-magnet brushless AC machines. *IEEE Trans. Magn.* **44**(12), 4659–4667 (2008).

[23] Qu, H. & Zhu, Z. Q. Analysis of split-tooth stator slot PM machine. *IEEE Trans. Ind. Electons.* **68**(11), 10580–10591 (2020).

[24] Amirkhani, M., Ghanbari, M. A., Kondelaji, M. A. J., Mirsalim, M. & Khorsandi, A. Performance analysis of outer rotor multi-tooth biased flux permanent magnet motors. *IEEE Trans. Energy Convers.* **38**(3), 1738–1752 (2023).

[25] Sriwannarat, W. et al. Structural multi-tooth modification of hybrid-excited doubly salient dual-pm machine for torque production improvement. *Appl. Sci.* **13**(3), 1414–1428 (2023).

[26] Amirkhani, M., Farahani, E. F., & Mirsalim, M. Study of An Improved Biased Flux Intermodular Permanent Magnet Motor. *IEEE Trans. Transp. Electrif*., (early access), (2023).

[27] Dutta, R., Chong, L. & Rahman, M. F. Design and experimental verification of an 18-slot/14-pole fractional-slot concentrated winding interior permanent magnet machine. *IEEE Trans. Energy Convers.* **28**(1), 181–190 (2012).

[28] Stockbrügger, J. O. & Ponick, B. Analytical determination of the slot and the end-winding portion of the winding-to-rotor capacitance for the prediction of shaft voltage in electrical machines. *Energies* **14**(1), 174–187 (2020).

[29] Dietz, A., Di Tommaso, A. O., Marignetti, F., Miceli, R. & Nevoloso, C. Enhanced flexible algorithm for the optimization of slot filling factors in electrical machines. *Energies* **13**(5), 1041–1062 (2020).

[30] Müller, K., Wanke, A., De Gersem, H., & Burkhardt, Y. Evaluation of tooth forces of skewing configurations of permanent magnet synchronous machines regarding rotor eccentricities in 2D and 3D FE simulations. In* IEEE ECCE Europe*, pp. 1–8 (2024).

[31] Kim, K., Hwang, M., Kim, H. K. & Cha, H. Torque improvement and magnetic flux leakage reduction in interior permanent magnet axial flux motors with flux barrier structure. *IEEE Access* **12**, 150869–150879 (2024).

[32] Carraro, E., Bianchi, N., Zhang, S. & Koch, M. Design and performance comparison of fractional slot concentrated winding spoke type synchronous motors with different slot-pole combinations. *IEEE Trans. Ind. Appl.* **54**(3), 2276–2284 (2018).

[33] Abdel Tawab, M. A., Shatla, H. & Bahy, M. Analysis of slotted tooth switched reluctance motors for electric vehicle applications. *J. Electr. Comput. Eng.* **1**, 449 (2024).

[34] Ibrahim, M., Masisi, L. & Pillay, P. Design of variable flux permanent-magnet machine for reduced inverter rating. *IEEE Trans. Ind. Appl.* **51**(5), 3666–3674 (2015).

[35] Ibrahim, M., Masisi, L. & Pillay, P. Design of variable-flux permanent-magnet machines using alnico magnets. *IEEE Trans. Ind. Appl.* **51**(6), 4482–4491 (2015).

[36] Hamby, D. M. A review of techniques for parameter sensitivity analysis of environmental models. *Environ. Monit. Assess.* **32**, 135–154 (1994). 24214086 10.1007/BF00547132

[37] Ning, S. et al. A novel double stator hybrid-excited flux reversal permanent magnet machine with halbach arrays for electric vehicle traction applications. *IEEE Access* **11**, 113255–113263 (2023).

[38] Chen, C. et al. Torque performance enhancement of flux-switching permanent magnet machines with dual sets of magnet arrangements. *IEEE Trans. Transp. Electrif.* **7**(4), 2623–2634 (2021).

[39] Almandoz, G., Gómez, I., Ugalde, G., Poza, J. & Escalada, A. J. Study of demagnetization risk in PM machines. *IEEE Trans. Ind. Appl.* **55**(4), 3490–3500 (2019).

[40] Li, H., Xia, C., Song, P., & Shi, T. Magnetic field analysis of a Halbach array PM spherical motor. *IEEE Inter. Conf. Auto. Logis.*2019–2023 (2007).

[41] Li, H., & Xia, C. Halbach array magnet and its application to PM spherical motor. *IEEE Inter. Conf. Electric. Mach. Sys. *3064–3069 (2008).

[42] Wu, Z. Z., Zhu, Z. Q. & Shi, J. T. Novel doubly salient permanent magnet machines with partitioned stator and iron pieces rotor. *IEEE Trans. Magn.* **51**(5), 1–12 (2015).26203196

